# Does love in the ivory tower fix the leaky pipeline? How academia’s homogamous relationships shape careers

**DOI:** 10.1371/journal.pone.0344105

**Published:** 2026-03-25

**Authors:** Antonia Velicu, Manon Fauvelais, Julia Jerke, Heiko Rauhut, Bruno Lemaitre

**Affiliations:** 1 Department of Sociology, University of Zurich, Zurich, Switzerland; 2 Global Health Institute, School of Life Sciences, Swiss Federal Institute of Technology-Lausanne (EPFL), Lausanne, Switzerland; 3 Swiss National Science Foundation, Bern, Switzerland; Instituto Tecnologico Autonomo de Mexico, MEXICO

## Abstract

The persistent “leaky pipeline”, i.e., women remaining underrepresented in advanced academic roles, often links to the adverse impact of parenthood on women’s careers compared to men’s. This study delves into how the struggle to balance academic success and family life might be pushing female scientists out of academia, and how the less-studied concept of homogamy—here, the forming of heterosexual relationships between individuals with the same profession—influences academic careers. Drawing on data from the 2021 “Academic career, partnership, and family” survey by swissuniversities, this research pursues two objectives: assessing whether homogamous partnerships help mitigating career challenges faced by mothers, and investigating the broader impact of homogamy on academic careers and work-life balance. The findings show that homogamy is common among Swiss scientists. Homogamous women, especially when their partner works in the same institution, perceive parenthood as posing fewer career obstacles. Conversely, male scientists in such relationships state the opposite. Additionally, homogamous couples report benefiting from stimulating discussions and partner support while encountering greater mobility constraints. This research offers insights into how homogamy affects academic careers, providing a nuanced understanding of how academics navigate their pursuit of successful careers alongside personal lives.

## 1 Introduction

In the world of academia, love and science often go hand in hand. The phenomenon of homogamy, or the tendency for individuals to form relationships with others who share a similar background and interests [1], has been observed in various occupational areas, but perhaps nowhere it is more prevalent or potentially influential than in academia [1–3]. Indeed, the unique circumstances of academic life, where research and teaching responsibilities often extend far beyond the traditional 9-to-5 workday, create an environment where the boundaries between personal and professional life can blur. This presents challenges, but it also creates opportunities for couples who share similar academic interests and goals to support each other both personally and professionally. As one scientist in a recent publication in Nature puts it, “*We wake up talking science and go to sleep talking science, but we wouldn’t have it any other way!*” [4]. This type of arrangement can cultivate a shared enthusiasm for knowledge-seeking, provide an arena for mutual feedback and support, and enable the exchange of critical information throughout the day and over the course of life [2,5].

At the same time academia’s competitive environment, characterized by demanding hours and international mobility, poses challenges for couples, especially when starting a family [6,7]. Women, in particular, face pronounced difficulties as academia inherently functions as a gendered organization, reinforcing hierarchical structures [8,9]. Mothers encounter additional hurdles [10], juggling childcare and domestic responsibilities, thereby constraining their career availability [11–13] and productivity [14]. Taking for example an anecdotal look at the very few female Nobel prize laureates in science (N = 22), only 68% of them had children (compared to more than 90% of the male laureates), and among those who have had children, many talk about the challenges of balancing a high achieving career and family. For instance, Tu Youyou, the 2015 Nobel Prize laureate in physiology or medicine has opened up about the sacrifice she made for her career: “*To focus on research, I left my younger daughter with my parents in Ningbo and sent my elder daughter to a full-time nursery [...] My younger daughter couldn’t recognize me when I visited my parents three years later, and my elder daughter hid behind her teacher when I picked her up upon returning to Beijing after a clinical investigation*.” [15]

Entering a homogamous heterosexual relationship may hold several potential benefits for women. According to research done outside of academia, dual-career couples, in which both partners hold high-ranked or high-demanding positions, tend to divide household chores more equally than traditional male-breadwinner households [16–18]. This can lead to greater equality and a more balanced distribution of responsibilities between partners, something that is especially beneficial to women. Homogamy may thus provide a key advantage for those who seek to excel in competitive scientific fields, especially for female scientists, raising the question: could homogamy mitigate the motherhood penalty?

We aim to address this gap in the literature by drawing on a recent large-scale data set from Switzerland [19,20]. The survey gives us the opportunity to examine homogamy’s impact on career success as perceived by researchers and disentangle ways in which it may be advantageous. We hypothesize that academic homogamy, understood as heterosexual partnerships in which both partners are active in academia, may benefit career advancement through two channels: (1) Homogamy could provide a benefit by being in a more equal partnership through mutual investment of the two partners regarding childcare and house duties. Considering the heavy burden of maternity, this could be especially advantageous for female scientists when children come into play. (2) As homogamous couples share academic experience, environment, and networks, we presume homogamy will positively affect their career.

The findings paint a vivid picture: Homogamy thrives among scientists in Switzerland. Further, we observe that female scientists indeed evaluate the impact of children on their careers much less negatively when they are in a homogamous heterosexual relationship, particularly when their partner works in the same academic institution. Interestingly, the opposite effect is observed among male scientists. Furthermore, as expected, homogamous participants report experiencing greater benefits from stimulating discussions and partner support, although they face greater mobility constraints compared to non-homogamous couples.

In contributing to the literature, our paper offers a nuanced perspective on the dynamics of homogamy within the academic realm. While initiatives aimed at promoting gender equality in academia often face challenges [21], our exploration of homogamous relationships highlights a potential avenue to naturally address some of the challenges women in science confront. By delving into the role and impact of homogamy on academic careers, especially for women, our research not only complements but also enriches the existing discourse on gender dynamics in academia.

## 2 Background

### 2.1 The starting point: Having children negatively affects the academic career of scientists

Academia is characterized by a highly competitive working environment that requires extensive working hours and international mobility, and, thus, creates a challenging environment for family formation [6,22]. Due to the still widely prevailing traditional family model, female scientists are often much more affected by these constraints than their male colleagues. In most cases, women are more involved in childcare and domestic work and can, therefore, be considered the primary caregiver [11,13]. At the extreme, this also leads to successful female academics being more likely to remain childless compared to their male colleagues and women with non-academic professions [23], since childless women face less obstacles when climbing up the career ladder [24]. As a result, men and childless women are overrepresented in tenured positions, while mothers are more likely to have a temporary or part-time position [25] or to drop out of academia completely. While it is well-documented that organizational academic structures seem to disadvantage women (e.g., [26,27]), recent research suggests that reasons for the leaky pipeline (the declining proportion of women in higher academic positions, [28] p.216) may also be found in the private support structures, the work-life-balance and priorities outside of academia of female scientists (e.g., [6]). In summary, a reason for female scientists dropping out of academia may lie in the difficulty of balancing a successful academic career with family life. We believe that reducing the motherhood penalty, i.e., the negative effect that childbearing has on women’s careers, could contribute to plugging the leaky pipeline, which is in line with previous research on postdocs [29]. Consequently, the private support structure of female scientists may likely play a crucial role in increasing the proportion of women in higher positions.

### 2.2 Introducing homogamy

The terms “collaborative couple” or “creative couple”, as they are commonly referred to in popular discourse, have direct roots in the scholarly construct of homogamy. Homogamy refers to relationships between individuals with similar backgrounds or characteristics, and has been broadly studied across various domains, including academia [1,30,31]. However, the term’s application in academic research often varies, with most studies discussing dual-earning or dual-career couples; meaning that both partners are gainfully employed but not necessarily that both have academic roles [32].

Keeping this in mind, research reveals a gendered trend in homogamy in academia, with a greater prevalence among women [30,31]. Notably, studies indicate that about 40% of women and 35% of men in U.S. universities are part of dual-career academic couples [33–35]. Additionally, as one moves up the academic hierarchy, the gender gap intensifies: 57% of women professors are in dual-career relationships compared to 49% of women in mid-rank positions [31], while the proportion of men in dual-career relationships remains the same (31% at both levels). This also underlines that the proportion of homogamous women increases with every step on the career ladder.

Given the diversity in definitions, it’s worth noting that our study focuses specifically on academic homogamy – heterosexual relationships where both individuals are academics. We argue that this subset of homogamous relationships is particularly relevant for a deeper understanding of gender dynamics and career advancement within academia. Being in such relationships often involves both partners sharing their resources, knowledge, network, and feedback on their respective work [36]. Homogamy thus seems to benefit career advancement for both partners, especially since it is very common in successful scientists [37,38]. Considering this, our study aims to delve deeper into academic homogamy to understand its unique benefits and its role in gender dynamics and career advancement within academia.

### 2.3 The impact of homogamy

Since having children often negatively affects the academic career of scientists, the question arises, whether academic homogamy might lead to a better compatibility of family and career. Previous work indicates that the flexibility provided by academic life indeed provides opportunities for collaborative couples to better arrange the presence of children and academic mobility [39,40]. It further shows that dual-career academic couples express having more egalitarian beliefs regarding partnerships [41–45]. The latter might particularly imply an increased paternal involvement and we, thus, expect that the effect of homogamous heterosexual relationships will differ for female and male scientists. Since the traditional family model still views mothers as primary caregivers, partnerships that share childcare and housework equally may particularly enable mothers to focus on their careers and lessen the “advantage” of fathers. Our study therefore investigates the following hypothesis: Being in a homogamous heterosexual relationship specifically mitigates the motherhood penalty that female scientists face.

### 2.4 Benefits of homogamy beyond parenthood

Besides our primary focus on the impact of homogamy in alleviating the motherhood penalty, we also believe it is important to consider their potential benefits beyond the realm of parenthood. Research suggests that homogamous partnerships are prevalent among successful scientists, which may indicate a broader utility for career progression [37,38]. Extensive qualitative interviews reveal that such relationships enable the sharing of valuable resources, such as knowledge and professional networks, that can be critical in competitive academic environments [2,5,36]. Additionally, partnerships between individuals with similar educational backgrounds can result in shared cultural values and mutual intellectual support, offering both professional and personal benefits [46].

We therefore see a need to further explore the broader implications of homogamous relationships in academia. Specifically, we are interested in how these relationships might equip scientists—especially women, who often face unique professional barriers—to better navigate the challenges of their academic careers.

## 3 Materials and methods

### 3.1 Data: The Academic Career, Partnership, and Family survey (ACPF)

In exploring our hypotheses, we use survey data from Switzerland, a country strategically located in the heart of Europe. Switzerland’s appeal is underscored by renowned technical institutions such as ETH Zurich, reflecting its status as a center for leading academic pursuits. Embracing a multicultural identity, Switzerland boasts several official languages, including French, Italian, German, and English, contributing to a diverse and inclusive academic environment. In addition, the country consistently ranks high in global quality of life indices, underscoring its appeal as a favorable environment for both professional and personal pursuits. Recognized as attractive employers for academics, Swiss universities offer notable family support mechanisms. Paid maternity leave is 14 weeks, with some institutions extending it to 16 weeks, while fathers or legal “other partners” benefit from leave ranging from two weeks to 20 working days, depending on the university’s policy. Notably, some universities provide on-campus childcare services, which complement broader family-friendly initiatives. In addition, the existence of dual career support programs exemplifies a commitment to addressing spousal career considerations through innovative hiring practices. However, the leaky pipeline phenomenon is also present, with women representing 43.4% of assistant positions, 33.2% of postdoctoral roles, and only 27.7% of professorships across all Swiss universities in 2022 [47].

The “Academic Career, Partnership and Family” survey (ACPF) specifically focuses on dual career couples (defined as couples in which both partners work at least at 80% and have at least a master’s degree) and examines among others the partnership structure, the impact of children and partnership on the academic career, and the division of domestic work and childcare [19,20]. It was conducted by swissuniversities in coordination with the École polytechnique fédérale de Lausanne (EPFL). The target group of the survey was all persons with an academic position at a Swiss university or in the ETH Domain and with a degree at master’s level or higher. A total of 40,798 people were contacted. The sample consists of 6067 scientists over all subjects from 12 Swiss universities (women: 53%; PhD students: 43%; Postdocs: 31%; Professors: 26%, see S1 Table in the Appendix for detailed sample description). The survey covers all academic disciplines, including humanities and social sciences, economics, law, natural and exact sciences, medicine, engineering, and interdisciplinary fields; accordingly, we use the terms “science” and “academia” interchangeably to refer to academic employment across disciplines [20]. The representativity analysis of the sample in S2 Table in the Appendix shows a slight overrepresentation of women and professors in the sample [20].

The survey included a question regarding the nature of the respondents’ relationship allowing us to monitor homogamy. We considered participants to be in a homogamous relationship if they answered “yes” to “Does your partner work in academia, too?”. We are also interested in whether the academic-geographic closeness plays a role when considering couples in science. We therefore distinguish three different degrees of homogamy: High closeness (“my partner is (mainly) employed at the same institute as I am”); medium closeness “my partner is (mainly) employed at another institute in Switzerland”); low closeness (“my partner is (mainly) employed at another institute outside of Switzerland”) with non-homogamous respondents as the reference group (partner is not in academia). Note that we exclude singles from our analysis. Although we specifically focus on homogamous gender differences, we also control for the professional status of the respondents (pre-doc, postdoc, and professor), whether their partner lives in the same household (yes/ no), and if necessary, how many hours they report working in a week (in percentages), and whether they have children (yes/ no). Control for these variables also mitigates the effects of overrepresentation of some demographic groups in our sample.

### 3.2 The negative and positive effects of parenthood

The ACPF survey contains comprehensive information about the partnership of the surveyed scientists such as the (academic) position of their partner, whether they have children and how they organize childcare, as well as how they share domestic work with their partner. As part of the survey, scientists with children (N = 2370) were further asked to rate a set of ten statements on the potential impact that having children has had on their career (see Table 1 for the exact phrasing). These assessments allow us to evaluate the subjectively perceived effect of parenthood on the academic career, and to estimate the differences between women and men as well as estimate the effect of being in a homogamous partnership. We perform a factor analysis on the item battery to identify latent factors underlying the statements. With a Kayser-Meyer-Olkin value of 0.83, the data is very well suited to perform a factor analysis. Also, the Bartlett test points in the same direction as it shows a significant value. A visualization of the factor analysis is provided in the Appendix. We use the statistical software R and the fa() command from the library psych 2.1.9 that provides among others factor analysis, principal component analysis, cluster analysis and reliability analysis. We use the minimum residual solution and perform a varimax rotation. The analysis suggests two factors (see S1 Fig in Appendix for the factor loadings), with the first one encompassing the negative impact of children on career progression (“children hinder career”). This factor summarizes various challenges related to time management, motivation, diminished support from the professional sphere, perceived setbacks in career advancement, reduced work hours, and decreased academic output. The second factor comprises the positive impact of children on professional growth (“children foster career”) which sums the positive influence on work efficiency and the ability to overcome professional challenges with greater ease. We will not discuss the factors in full detail here and instead provide an overview of the resulting factors in S1 Fig in the Appendix. Table 1 shows the mean values and the confidence intervals of the factors divided for women and men. All values are higher for women, except for the one that states there was no influence, which suggests that women are more affected by children. In what follows, we will build on the two determined factors and analyze the influence that a homogamous relationship has on the impact of children on the academic career. In a second step, we further introduce the interaction between homogamy and gender (0 – male, 1 – female).

**Table 1:** Statements on the impact that having children has had on the career.

Statement	Female mean (± CI)	Male mean (± CI)	Factor
I make more efficient use of my time at work since I’ve had children.	3.01 (± 0.21)	2.79 (± 0.20)	children *foster* career
Since having children, I am able to overcome professional challenges with greater ease.	2.57 (± 0.07)	2.49 (± 0.07)	
I am certain I would be farther along in my career had I not had children.	3.02 (± 0.67)	2.33 (± 0.67)	children *hinder* career
After I had children, I wanted / had to greatly reduce my hours at work.	2.94 (± 0.31)	2.62 (± 0.31)	
Since having children, I publish less.	2.68 (± 0.41)	2.26 (± 0.41)	
After I had children, my professional environment no longer took my career plans as seriously or did not support me as well as they had previously.	2.58 (± 0.63)	1.61 (± 0.63)	
Attempting to combine children and career has put a large strain on my current partnership.	2.53 (± 0.23	2.29 (± 0.23)	
My academic work has not reached the quality it had prior to having children.	2.17 (± 0.22)	1.95 (± 0.22)	
The fact that I have children has had neither a positive nor negative influence on my career.	2.07 (± 0.43)	2.50 (± 0.07)	
I had to fight to motivate myself at work after I had children.	1.95 (± 0.13)	1.81 (± 0.13)	

Note: The statements were measured on a four point Likert scale that we reversed so that the scale goes from 1 - “I absolutely disagree” to 4 - “I absolutely agree” and are listed in a decreasing order within the factors. To define the factors, we were guided by an exploratory factor analysis. We also show the mean value and the confidence interval differentiated for women and men.

### 3.3 The effect of having a homogamous relationship on the career

The ACPF survey further provides an item battery with nine statements that asked scientists currently in a relationship (N = 4731) about the impact of their heterosexual relationship on their career (for example: “Discussions with my partner are stimulating for my academic work” or “I am less mobile than is advantageous to my career due to my partner’s career”, see Table 2). We again perform a factor analysis to identify latent factors underlying the statements. With a Kayser-Meyer-Olkin value of 0.65, the data is suited to perform a factor analysis. Except for the last item, the individual variables show relatively high MSA values (0.6 to 0.74), which means that they are also individually well suited to identify factors. We test Bartlett and see it is significant ($\chi^{2}\;$ = 155, p < 0.000). According to the eigenvalues a two- or three-factor solution seems reasonable. However, we also evaluate the interpretation of different factor solutions and eventually decide for a four-factor solution. The analysis suggests four factors (see S2 Fig in Appendix for the factor loadings): relationship intellectually stimulates career (partner’s discussions stimulate academic work, and their professional network benefits career), relationship generally supports career (partner supports in difficult professional situations and provides valuable career advice), relationship constraints mobility (lack of mobility hampers career advancement, and it is challenging for both partners to have meaningful careers in the same location), and career strains relationship (the demands of work and pursuing successful careers are straining the relationship). For factor 1 and 2, high values imply a positive influence of having a partner on the career, and for factor 3 and 4 high values imply a negative influence of the partnership on the career or vice versa. Table 2 also shows the mean values and the confidence intervals of the factors divided for women and men. We assume that partner’s general support is relevant for all scientists in relationships while intellectual stimulation might be particularly relevant for scientists in homogamous relationships. We also assume that homogamous relationships report higher constraints in regard to mobility.

**Table 2:** Statements on the impact that the partnership has had on the career.

Statement	Female mean (± CI)	Male mean (± CI)	Factor
Discussions with my partner are stimulating for my academic work.	3.11 (± 0.27)	2.84 (± 0.27)	Relationship intellectually stimulates career
I profit from my partner’s professional network.	1.75 (± 0.04)	1.70 (± 0.04)	
My partner has supported me in difficult professional situations.	3.53 (± 0.03)	3.50 (± 0.03)	Relationship generally supports career
My partner’s advice has led me to making the right career decisions.	3.05 (± 0.04)	3.01 (± 0.04)	
It is unlikely that my partner and I can both pursue meaningful careers and live in the same place.	2.57 (± 0.08)	2.48 (± 0.08)	Relationship constraints mobility
I am less mobile than is advantageous to my career due to my partner’s career.	2.43 (± 0.01)	2.42 (± 0.01)	
Both of us pursuing a successful career has placed a big strain on our relationship.	2.84 (± 0.27)	2.39 (± 0.04)	Career strains relationship
The demands my work places on my time puts a big strain on our relationship.	2.40 (± 0.05)	2.46 (± 0.05)	
I am able to pursue my professional goals because my partner has assumed most of the household and caring duties.	1.77 (± 0.37)	2.15 (± 0.37)	(*)

Note: The statements were measured on a four point Likert scale that we reversed so that the scale goes from 1 - “I absolutely disagree” to 4 - “I absolutely agree” and are listed in a decreasing order within the factors. To define the factors, we were guided by an exploratory factor analysis. We also show the mean value and the confidence interval differentiated for women and men. (*) We excluded this item from the subsequent analyses since it had very low factor loadings to all of the factors.

## 4. Results

### 4.1. When climbing up the hierarchy, women have more often homogamous relationships

**Fig 1:**
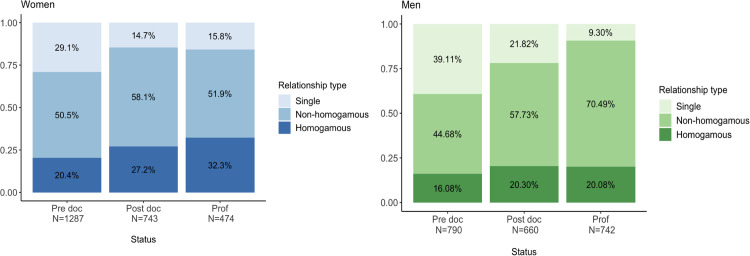
Stacked bar plot of the relationship type of the respondents. The bars are stacked by the relationship type (proportion of single, homogamous and non-homogamous) and split by gender and professional status. The figure is based on the ACPF survey data.

Before we look at the role of homogamy in the impact of children on academic careers, we first want to assess the prevalence of homogamy in our sample. The data reveals a gender disparity with women in heterosexual relationships generally being more homogamous than men (from all the scientists that are in heterosexual relationships, 29.95% of the women are homogamous vs. 23.95% of the men; z-score = 5.15, p-value < 0.00). Further, Fig 1 also reveals that homogamy becomes more prevalent for both men and women as they climb the hierarchy, and that this rise is particularly marked for women (11.84% increase between the PhD and professor level for women versus 4% for men). Of note, our analysis reveals that the proportion of single men decreases when climbing the hierarchy, a tendency less pronounced for women.

### 4.2. Homogamy mitigates the motherhood penalty

Does homogamy influence the career advancement of mothers? In order to explore this, we start with reducing the sample to parents only. First, descriptively comparing the factor scores by gender, we find that women have a higher average value for both children scores. They more strongly agreed with the statements about the adverse effects of children on career advancement (factor “children hinder career”, mean factor scores: 0.304 for women vs −0.298 for men, t-test: t = −13.123, df = 1467.2, p-value < 0.000). This means that they on average report suffering more from challenges related to time management, motivation, diminished support from the professional sphere, perceived setbacks in career advancement, reduced work hours, and decreased academic output. At the same time women report slightly higher positive effects of motherhood on professional growth (factor “children foster career”, mean factor scores: 0.067 for women vs −0.073 for men, t-test: t = −3.6199, df = 1441.8, p-value < 0.000), but the group differences are small.

Second, we conduct a set of linear regression analyses with the two factor scores as dependent variables: the negative influence of having children on career progression and positive effects of parenthood on professional growth. In a first step, we explore whether there are general differences between homogamous and heterogamous relationships and between women and men. We control for the professional status of the respondents, how many hours they report working in a week and whether their partner lives in the same household, resulting in a sample size of N = 864 (see Table 3). The results in column (1) and (3) support the descriptive observation from before: women have significantly higher scores both for the negative and the positive impact of children on the career (coeff = 0.381, p < 0.01 for the negative impact, and coeff = 0.189, p < 0.01 for the positive impact). This suggests that female scientists perceive the positive aspects of having children as much more positive and at the same time the negative aspects as much more negative than their male counterparts. In contrast, there is no difference between scientists in homogamous and non-homogamous relationships on the aggregate level.

**Table 3:** Results of the linear regression analyses of children hindering or fostering career.

	children hinder career		children foster career	
	(1)	(2)	(3)	(4)
Homogamous	−0.035 (0.066)	0.134 (0.098)	−0.066 (0.058)	−0.083 (0.085)
Female	0.381*** (0.063)	0.477*** (0.075)	0.189*** (0.055)	0.179*** (0.066)
Homogamous*female		−0.307** (0.130)		0.030 (0.114)
Co-living	−0.176 (0.116)	−0.165 (0.116)	0.096 (0.101)	0.095 (0.101)
Postdoc	0.046 (0.118)	0.069 (0.118)	−0.117 (0.103)	−0.119 (0.103)
Prof	−0.559*** (0.116)	−0.524*** (0.117)	−0.031 (0.101)	−0.034 (0.102)
Worktime in %	0.009*** (0.002)	0.009*** (0.002)	−0.003 (0.002)	−0.003 (0.002)
Constant	−0.124 (0.176)	−0.190 (0.178)	0.021 (0.154)	0.028 (0.156)
Observations	864	864	864	864
R2	0.204	0.209	0.019	0.019
Adjusted R2	0.199	0.203	0.012	0.011

Note: that the factors are standardized and are therefore on a standard normal scale. For factor 1 high values imply a negative effect of children on the career (models 1 & 2) and for factor 2, high values imply a positive effect of children on the career (models 3 & 4). ***p<0.1 ; **p<0.05 ; *p<0.01 ; Standard errors in parentheses.

**Fig 2:**
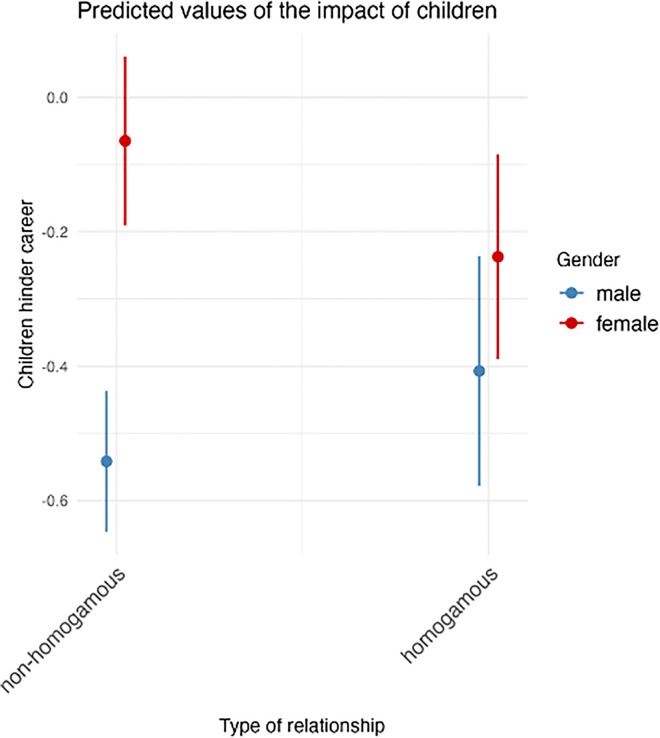
Marginal effects of interaction term. The graph plots the predicted values of the children’s impact on career based on the ACPF item battery completed by scientists with children (N = 864), stratified by gender and relationship type. This figure represents the marginal effects of the interaction term, with covariates held constant. These predictions are for a professor that lives in the same household as their partner and works 42.48h per week.

When looking at the effect of homogamy for women and men separately (see columns (2) and (4)) which include an interaction of gender and homogamy), we observe notable differences. While male scientists report larger agreement with the negative impact of children on their career when being in a homogamous relationship (coeff = 0.134, not significant), being in a homogamous relationship comparatively reduces the perceived negative impact of children for female scientists (interaction coeff = −0.307, p<0.05 ). To underline the differences between women and men, we visualize the predicted values in Fig 2. Whereas there is a notable disparity between the predictions for heterogamous men and women, there seems to be some kind of assimilation between women and men in homogamous relationships suggesting a balancing out pattern. When split by the degree of closeness of homogamy, we see that actually only women in relationships with a scientist from the same institute report being less negatively affected by having children (S3 Table in the Appendix, linear regression, coeff = −0.457, SE = 0.185, p<0.01 , N = 847). The other degrees of homogamy are not statistically significant compared to non homogamous relationships.

In contrast, we do not see differences in the effect of a homogamous relationship between women and men for the positive impact of children on their career (main coeff = −0.083; not significant, interaction coeff = 0.030, not significant). Neither female nor male scientists, thus, seem to benefit from homogamy in this aspect.

### 4.3. Childcare duties are shared more equally in homogamous relationships

**Fig 3:**
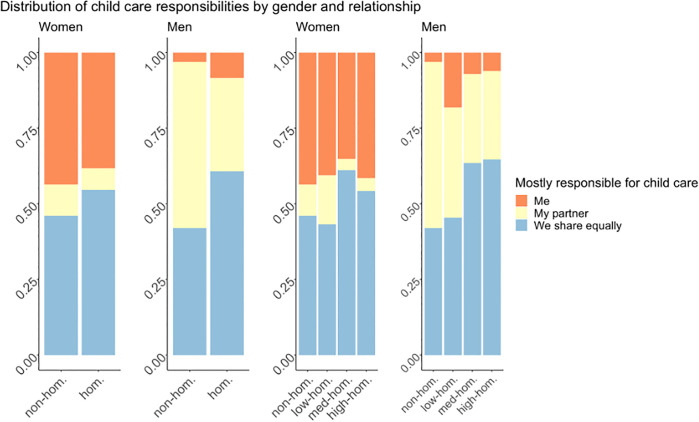
Stacked bar plot of the distribution of childcare responsibilities. This figure displays the distribution of childcare responsibilities, the first two subplots are categorized by gender and relationship type and subplots 3 and 4 by gender and closeness of homogamy. The color-coded bars within each subplot depict the proportion of individuals who perceive themselves as primarily responsible for children (labeled “me” and colored orange), those who attribute this responsibility to their partners (labeled “my partner” and colored yellow), and those who share child care responsibilities equally (labeled “we share equally” and colored blue).

Is it possible that homogamous couples have a more balanced distribution of childcare duties, and, thus, a more equal sharing of the burdens of parenthood, which in turn allows both partners to devote as much time and energy to their careers? To address this question, we examine further how care responsibilities are distributed within homogamous and heterogamous couples (Fig 3). Notably, more homogamous female scientists (55%) than non-homogamous female scientists (46%) report that they equally share the responsibilities with their partner. This difference is even larger for male scientists: among those within homogamous relationships, 61% state that they equally share childcare whereas the share is only 42% in non-homogamous relationships (first two subplots of Fig 3). Thus, even though female scientists in general report allocating more time to domestic work and childcare, men claim to contribute relatively more to these tasks in homogamous relationships. This is particularly noticeable within higher degrees of homogamy (last two subplots of Fig 3).

The previous results suggest that homogamy benefits women by cushioning the negative impact that children have on their career, and that there is a tendency towards a more equal distribution of childcare responsibilities in homogamous relationships. This finding provides valuable insights into the dynamics of these relationships and serves as a foundation for comprehending how homogamy may influence careers in academia.

### 4.4. The effect of homogamy on career and academic support

We explore the effect of homogamy also on couples independent of whether they have children or not. We conduct multiple linear regression analyses with the factor scores of work demands and relationship strain, intellectual stimulation and professional support, partner’s support and career guidance, and mobility constraints as dependent variables (see Table 4). We again specifically focus on gender and differences with respect to the relationship type, but we also control for the status of the respondents, whether they have children, and whether the partner lives in the same household. In the first set of regressions, we treat homogamy as a binary variable and distinguish between scientists who have a partner inside academia and scientists who have a partner outside academia. In a second step, we also consider the different degrees of homogamy (S3 Table in Appendix).

**Table 4:** Results of the linear regression analyses of the academic support and constraints factors.

	Relationship intellectually stimulates career	Relationship generally supports career	Relationship constraints mobility	Career strains relationship
	(1)	(2)	(3)	(4)
Homogamous	0.751*** (0.039)	−0.029 (0.036)	0.168*** (0.031)	0.008 (0.042)
Female	0.235*** (0.036)	0.023 (0.034)	0.00003 (0.029)	0.024 (0.038)
Children	−0.021 (0.040)	−0.111*** (0.037)	0.086*** (0.032)	0.219*** (0.043)
Co-living	0.103** (0.050)	0.187*** (0.047)	−0.159*** (0.040)	−0.345*** (0.053)
Postdoc	0.063 (0.045)	0.029 (0.042)	0.129*** (0.036)	0.155*** (0.048)
Prof	0.122** (0.048)	−0.014 (0.045)	−0.288*** (0.039)	0.040 (0.052)
Constant	−0.452*** (0.055)	−0.066 (0.051)	0.109** (0.044)	0.162*** (0.059)
Obs.	2,042	2,042	2,042	2,042
R2	0.182	0.012	0.085	0.036
Adjusted R2	0.180	0.009	0.082	0.033

Note: *** p<0.1 ; ** p<0.05 ; * p<0.01 ; Standard errors in parentheses.

As expected, homogamous participants report significantly more benefits from stimulating discussions and partner support than non-homogamous ones (model 1, coeff = 0.751, SE = 0.039, p<0.001 , N = 2042). Based on these self-reported perceptions, it thus seems that forming a collaborative couple benefits both partners’ careers through mutual support, networking, and stimulating discussions. However, homogamous respondents also report having more difficulties balancing their demanding careers and their relationship and dealing with mobility constraints (model 3, coeff = 0.168, SE = 0.031, p<0.001 , N = 2042), hinting that this kind of partnership is not without its own challenges. Both homogamous and non-homogamous couples encounter comparable levels of work-related strain in their relationships (model 4), and there are no significant differences in terms of general support (models 2), as homogamy does not appear to affect the career guidance provided by partners in professional situations. Interestingly, there are no or only small differences between women and men in the impact of the relationship on the career. Sole exception is the intellectual stimulation from the partner, which women perceive to be significantly higher than men (model 1, coeff = 0.235, p<0.01 ). Further, there are no significant interaction effects between gender and homogamy (see S5 Table in the Appendix for results).

We observe several noteworthy patterns with respect to the covariates. The presence of children seems to decrease the perceived positive impact of the relationship significantly and, at the same time, increase the negative impact of the relationship – all models, except the first, show a significant effect (p<0.01 ). The effects are of exactly opposite directions when it comes to sharing a household: scientists living together with their partner perceive the positive impact of the relationship significantly higher and the negative impact significantly lower (p<0.05  for all models). In summary, scientists without children and scientists sharing the same household with their partner report significantly more benefits and fewer downsides of their partnership in terms of pursuing a career.

Taking a look at differences between homogamy degrees, the findings align with our previous results (see S3 Table in the Appendix): Homogamy continues to play a significant role in intellectual stimulation and professional support and mobility constraints. It appears that the closer the homogamous partner is, the greater the positive impact on work. Mobility constraints increase when partners belong to the same or a different but still national (Swiss) institute (compared to non-homogamous scientists).

## 5 Discussion

In scientific history, collaborative couples like Pierre and Marie Curie stand out, illuminating the potential synergies that can arise between partners in academia. Their partnership transcended the boundaries of their personal life, enabling groundbreaking discoveries in physics and chemistry. However, such collaborations raise an interesting question: To what extent do homogamous heterosexual relationships influence the trajectory of academic careers and the delicate act of balancing professional aspirations with familial responsibilities? The phenomenon known as the “leaky pipeline” remains a haunting narrative in academia, with a declining proportion of women ascending to higher career stages [28]. This decline is worsened by the so-called “motherhood penalty” where motherhood poses a significant obstacle in women’s academic careers, casting a shadow on their professional prospects [48,49]. The realm of homogamy therefore presents a unique lens through which we can examine these dynamics and possibly uncover strategies to remedy such systemic challenges. Drawing on a large-scale survey conducted by swissuniversities and EPFL among scientists in Switzerland, we investigated the implications of homogamous heterosexual relationships for the academic couple’s careers and their work-life balance.

### 5.1 High prevalence of homogamy, especially amongst female professors

While our sample overrepresents female professors compared to the Swiss academics population, it is consistent with past studies on the prevalence of dual career academic couples in US universities [33,34,38,50,51]. We observed homogamy rates of roughly 30% among women and 25% among men, slightly below findings from US studies [33–35]. It also confirms that female academics are more likely to be in a homogamous relationship compared to men [33,34,50]. Factors like the growing number of women in science could account for this high prevalence [51,52], alongside the career benefits it offers [34,42]. Interestingly, homogamy’s prevalence increases with rank among female academics, a trend inconsistent for males [30,31], suggesting its varying significance between genders. Considering our sample’s gender bias, further balanced studies are essential for a clearer understanding.

### 5.2 Homogamy may mitigate the reported motherhood penalty

Based on our results, we discovered that homogamous female academics – particularly those whose partners work at the same institution – report that childbearing has less of a negative impact on their career compared to their heterogamous colleagues. This coincides with reports from homogamous female faculty members that being with someone who knows and understands the academic world facilitates integrating and balancing the demands of having children with work [2]. For male scientists, in contrast, we see the exact opposite pattern: they report being more negatively impacted by family life with children when their partner is in academia as well. This effect could in part be explained by the fact that homogamous male scientists report sharing house chores more equally than their non-homogamous counterparts, as our findings show. Thus, our analysis concludes that collaborative couples have a less traditional family model defined by more equal distribution of childcare between parents, which is in line with previous work showing that dual-career academic couples express having more egalitarian beliefs regarding partnerships [41–45]. It is likely that the flexibility provided by academic life provides opportunities for collaborative couples to adjust the presence of children and mobility better [39,40], with a stronger paternal investment. Additionally, having a partner who is working in a similar academic environment can foster a deeper understanding and empathy for the challenges women scientists face. This shared understanding can lead to more effective communication and support between partners, reducing potential conflicts and facilitating the development of strategies to mitigate the negative impact of parenthood on their careers. Moreover, the closer the homogamous partner is, the more it mitigates the motherhood penalty. This may be explained by the fact that if they work at the same institute they live together and it is therefore easier to coordinate schedules and share responsibilities related to childcare and household tasks (as seen above). Also, institutes that employ both partners may have family-friendly policies in place, such as flexible work arrangements, on-site childcare facilities, or supportive leave policies. However, given the cross-sectional nature of the data, our findings should be interpreted as associational rather than causal.

### 5.3 Homogamy, cohabitation, and career stages

According to the self-reported perceptions from the ACPF survey, homogamous scientists report receiving greater professional benefits from their spouses than their heterogamous counterparts. Indeed, having a partner who understands the academic lifestyle is a critical source of support for academic couples [2,5]. Notably, we observed that cohabitation appears to accentuate the benefits of intellectual discourse and mutual professional support and be a buffer against work-related strains on relationships. This effect is magnified in the absence of children. Professors appear to draw significant advantages, possibly because they share similar career paths with their partners, which leads to a better mutual understanding. Moreover, women distinctly report benefiting more from intellectual interactions and support within relationships. This observation hints at a possible heightened value or sensitivity that women may place on these facets of partnership, or it could suggest a transfer of resources from men to women. Despite the professional advantages of homogamous relationships, they present unique challenges. Homogamous participants noted struggles in juggling demanding careers and relationships. Women, however, seemed less affected by the necessary professional concessions. Postdoctoral researchers and scientists with children face pronounced work strains compared to PhD-level counterparts, possibly due to escalated postdoc responsibilities combined with parenting challenges.

All in all, the role of homogamy cannot be understated. Despite the challenges, the size of the reported benefits suggests that the advantages of homogamous relationships outweigh the disadvantages, at least among our sample of scientists. However, further research should investigate these findings in larger and more diverse samples to fully understand the potential trade-offs of homogamous relationships in the scientific world.

The implications of our research on homogamous relationships in academia go beyond advocating for an academic dating service or positioning conferences as preferred dating platforms. Instead, dual-career support programs should be further developed, including resources for navigating the academic job market, with the goal of attracting and retaining talented individuals. We also propose an alternative interpretation of our findings that emphasizes the importance of both psychosocial and professional support. We now suggest that these need not to stem from the same source. While general emotional support and career guidance appear to be available across relationship types, professional stimulation and access to academic networks are reported primarily by homogamous couples. This pattern suggests that institutions could help compensate for these disparities by fostering professional exchange and network-building opportunities beyond intimate partnerships.

### 5.4 Limitations

In this paper, we suggest that homogamy might carry greater significance for women’s success. However, the underlying mechanisms remain multifaceted and not easy to disentangle. Without longitudinal data, we cannot definitively ascertain if homogamy attracts women to academia, aids in their retention, or is perhaps a byproduct of shared passion. It also poses the question whether these patterns echo traditional biases in partner selection, such as women seeking partners of higher status and men valuing caregiving qualities [53–55]. Given the cross-sectional design, we also cannot distinguish the direction and disentangle potential causal effects from selection processes: homogamous relationships may facilitate career persistence, but it is also possible that women who remain in academia are more likely to form partnerships with other academics. Further, longitudinal studies are therefore needed to determine how exactly homogamy impacts career progression.

It should be noted that the results, particularly those pertaining to women professors, may not be directly generalizable to broader academic populations due to the sample’s composition: there is a slight overrepresentation of women and professors in our sample. In essence, while the overrepresentation is a limitation in terms of generalizability, it offered us a unique opportunity to delve deeply into the experiences and perspectives of women professors regarding the challenges and dynamics of homogamy in Swiss academia. Moreover, this overrepresentation may highlight the very issue at hand, where women professors are particularly invested in understanding and addressing the factors contributing to the leaky pipeline. Further studies with more balanced gender representations are warranted.

Another limitation of our data is that we were unable to evaluate whether the couples in our analysis work in the same field, as collaborative pairs do. A previous study in 13 US research universities showed that over 60% of academic couples work in the same discipline [34], showing that it is an important factor to consider. Thus, homogamy most likely has a broader impact when both partners work in the same field or even on the same topic. Future investigations should analyze the impact of homogamy on career success considering different degrees of homogamy.

Lastly, we focused solely on gender dynamics in homogamous heterosexual relationships within academia, and while we include insights into family dynamics, we acknowledge that we overlooked other potential intersections (e.g., race or sexual orientation). Future research should aim to examine these intersections, e.g., whether and how these patterns differ in queer partnerships, offering a more comprehensive understanding of challenges and opportunities in academic environments. Our study lays the groundwork, urging subsequent research to explore the multifaceted dimensions of diversity and identity.

## 6 Conclusion

Navigating the challenges of academia is often harder for women, a situation commonly referred to as the *leaky pipeline*. Our study of Swiss academia has shown some interesting patterns related to this. Homogamy, or partnerships between those with similar educational backgrounds, is common, especially among women. These relationships are not just numbers; they also appear instrumental in distributing childcare more equitably, particularly benefiting women by mitigating the children penalty. Furthermore, participants in such relationships expressed strengthened mutual understanding and support, especially when working closer. However, this dynamic is not devoid of challenges. Mobility issues, particularly when children are involved, pose a tangible constraint. Despite these challenges, the positive impact on partnership dynamics and career support emphasizes the valuable role that homogamy plays in the realm of professional and personal aspirations.

Looking back, partnerships like those between Marie and Pierre Curie show the potential of such relationships. These couples not only achieved great academic success but also managed their personal and professional lives together effectively. In today’s academic world, understanding homogamous partnerships might be more important than ever. Recognizing the strengths and challenges of these relationships can help promote innovation, combine individual and shared goals, and perhaps offer a new way to view the challenges women face in academia.

Still, we must treat our results with caution. While our findings emphasize the positive aspects of homogamy in academia, it should not be misconstrued to mean that homogamous partnerships are the only path for women’s academic progression. It’s more nuanced than that. But understanding the role of such partnerships can offer fresh perspectives, potentially fostering environments that celebrate creativity and collaboration. However, it’s essential to recognize that high levels of educational homogamy might lead to a lack of diffusion of scientific knowledge into broader society. [56] suggested that academic elitism could reinforce social hierarchies and reduce science’s accessibility to the general population. Moreover, [3] highlighted that educational homogamy can exacerbate income inequality, suggesting further societal divides. In sum, the deeper we delve into understanding the facets of homogamous partnerships, the closer we might come to enhancing scientific collaboration, creativity, and, ultimately, success while being acutely aware of its broader societal implications.

## Supporting information

S1 Table Sample description of the ACPF data. (PDF)

S2 Table Representativity of status groups and gender. (PDF)

S3 Table Results of the linear regression analyses of parenthood factors. (PDF)

S4 Table Results of the linear regression analyses including interaction effects. (PDF)

S5 Table Results of the linear regression analyses analyses of academic support and constraints factors. (PDF)

S1 Fig Factor analysis diagram of the impact of children. (TIFF)

S2 Fig Factor analysis diagram of impact of relationship. (TIFF)
