# Supplementary material for: Does love in the ivory tower fix the leaky pipeline? How academia’s homogamous relationships shape careers
Source: PLoS One. 2026 Mar 25;21(3):e0344105. doi: 10.1371/journal.pone.0344105 (PMC13016316; doi:10.1371/journal.pone.0344105)
Supplement: S1 Table — (PDF) [file pone.0344105.s001.pdf]

Table S1. Sample description of the ACPF data.

	N	%		N	%
**Gender**			**Children**		
Male	2769	46.3	No	3665	60.7
Female	3207	53.7	Yes	2368	39.3
**Professional Status**			**Cohabitation with partner**		
Pre-doc	2106	44.2	No	816	17.3
Post-Doc	1419	29.8	Yes	3893	82.7
Professors	1242	26.1	**Degree of homogamy**		
**Relationship status**			No homogamy	3456	75.1
Single	1336	22	Low Closeness	247	5.4
Non-homogamous	3456	57	Medium Closeness	439	9.5
Homogamous	1272	21	High Closeness	457	9.9

	N	mean	sd	min	max
Work time	3956	42.48	14.14	0	230
