# Supplementary material for: Does love in the ivory tower fix the leaky pipeline? How academia’s homogamous relationships shape careers
Source: PLoS One. 2026 Mar 25;21(3):e0344105. doi: 10.1371/journal.pone.0344105 (PMC13016316; doi:10.1371/journal.pone.0344105)
Supplement: S2 Table — (PDF) [file pone.0344105.s002.pdf]

Table S2. Representativity of status groups and gender.

	Switzerland (2021)[1]	ACPF (2021)[2]	Difference
**By status groups**		6,655 (100 %)	
Junior and senior academics	45,008 (90.60 %)	5,346 (80.33 %)	
Professors	4,669 (09.40 %)	1,309 (19.67 %)	+ 10.27%
**By gender**		6,556[3] (100 %)	
Male academic staff	29,390 (59.16 %)	3,116 (47.54 %)	
Female academic staff	20,287 (40.84 %)	3,440 (52.46 %)	+ 11.62%
**Total academic staff**	49,677 (100 %)		

[1] *Retrieved from the Swiss Federal Statistical Office (Bundesamt für Statistik BFS) 2022;*
[2] *Retrieved from Liechti et al. 2022a (p. 2);*
[3] *The total number differs from the status group, because 35 do not identify with the two categories and 64 did not answer the question.*
