# Supplementary material for: Does love in the ivory tower fix the leaky pipeline? How academia’s homogamous relationships shape careers
Source: PLoS One. 2026 Mar 25;21(3):e0344105. doi: 10.1371/journal.pone.0344105 (PMC13016316; doi:10.1371/journal.pone.0344105)
Supplement: S3 Table — (PDF) [file pone.0344105.s003.pdf]

Table S3. Results of the linear regression analyses of parenthood factors.

	Negative influence of children on career progression		Positive effects of parenthood on professional growth	
High closeness	-0.042<br>(0.246)	-0.174<br>(0.155)	-0.188<br>(0.214)	-0.079
Medium closeness	0.128<br>(0.152)	-0.024<br>(0.095)	-0.061<br>(0.133)	0.189***<br>(0.055)
Low closeness	0.209<br>(0.134)	-0.025<br>(0.094)	-0.040<br>(0.116)	-0.017<br>(0.082)
Female	0.481***<br>(0.076)	0.378***<br>(0.065)	0.177***<br>(0.066)	0.183***<br>(0.056)
High c. * female	-0.228<br>(0.308)		0.174<br>(0.268)	
Medium c. * female	-0.261<br>(0.192)		-0.056<br>(0.167)	
Low c. * female	-0.457**<br>(0.185)		0.046<br>(0.161)	
Live in partner	-0.176<br>(0.122)	-0.192<br>(0.122)	0.066<br>(0.106)	0.071<br>(0.106)
Postdoc	0.063<br>(0.119)	0.041<br>(0.119)	-0.125<br>(0.104)	-0.123<br>(0.104)
Prof	-0.523***<br>(0.118)	-0.560***<br>(0.118)	-0.052<br>(0.103)	-0.048<br>(0.102)
Worktime in %	0.008***<br>(0.002)	0.009***<br>(0.002)	-0.003<br>(0.002)	-0.003<br>(0.002)
Constant	-0.171<br>(0.184)	-0.100<br>(0.181)	0.073<br>(0.16o)	0.060<br>(0.157)
Observations	847	847	847	847
R^2	0.208	0.201	0.019	0.018
Adjusted R^2	0.198	0.194	0.006	0.009

*Note:****$p < 0.1$; **$p < 0.05$; *$p < 0.01$; Standard errors in parentheses.*
