# Supplementary material for: Does love in the ivory tower fix the leaky pipeline? How academia’s homogamous relationships shape careers
Source: PLoS One. 2026 Mar 25;21(3):e0344105. doi: 10.1371/journal.pone.0344105 (PMC13016316; doi:10.1371/journal.pone.0344105)
Supplement: S4 Table — (PDF) [file pone.0344105.s004.pdf]

Table S4. Results of the linear regression analyses including interaction effects.

	work demands and relationship strain		intellectual stimulation and professional support		partner's support and career guidance		mobility constraints	
	(1)	(2)	(3)	(4)	(5)	(6)	(7)	(8)
Homogamous	0.008	0.054	0.751***	0.733***	-0.029	-0.073	0.168***	0.224**
	(0.042)	(0.063)	(0.039)	(0.059)	(0.036)	(0.055)	(0.031)	(0.047)
Female	0.024	0.048	0.235***	0.225***	0.023	0.0001	0.00003	0.029
	(0.038)	(0.046)	(0.036)	(0.043)	(0.034)	(0.040)	(0.029)	(0.034)
Children	0.219***	0.218***	-0.021	-0.020	-0.111***	-0.110***	0.086***	0.084***
	(0.043)	(0.043)	(0.040)	(0.040)	(0.037)	(0.037)	(0.032)	(0.032)
Homogamous * female		-0.080		0.033		0.078		-0.100
		(0.084)		(0.078)		(0.073)		(0.063)
Co-living	-0.345***	-0.345***	0.103**	0.103**	0.187***	0.187***	-0.159***	-0.159***
	(0.053)	(0.053)	(0.050)	(0.050)	(0.047)	(0.047)	(0.040)	(0.040)
Postdoc	0.155***	0.157***	0.063	0.062	0.029	-0.031	0.129***	0.132***
	(0.048)	(0.048)	(0.045)	(0.045)	(0.042)	(0.042)	(0.036)	(0.036)
Prof	0.040	0.045	0.122**	0.121**	-0.014	-0.018	-0.288***	-0.282***
	(0.052)	(0.052)	(0.048)	(0.049)	(0.045)	(0.045)	(0.039)	(0.039)
Constant	0.162***	0.148**	-0.452***	-0.446***	-0.066	-0.052	0.109**	0.092**
	(0.059)	(0.060)	(0.055)	(0.056)	(0.051)	(0.053)	(0.044)	(0.045)
Observations	2,042	2,042	2,042	2,042	2,042	2,042	2,042	2,042
R^2	0.036	0.037	0.182	0.182	0.012	0.013	0.085	0.086
Adjusted R^2	0.033	0.033	0.180	0.179	0.009	0.009	0.082	0.083

*Note: This table is similar to Table 4; ***$p < 0.1$; **$p < 0.05$; *$p < 0.01$; Standard errors in parentheses.*
