# Supplementary material for: Does love in the ivory tower fix the leaky pipeline? How academia’s homogamous relationships shape careers
Source: PLoS One. 2026 Mar 25;21(3):e0344105. doi: 10.1371/journal.pone.0344105 (PMC13016316; doi:10.1371/journal.pone.0344105)
Supplement: S5 Table — (PDF) [file pone.0344105.s005.pdf]

**Table S5.** Results of the linear regression analyses of academic support and constraints factors.

| | work demands and relationship strain | | intellectual stimulation and professional support | | partner's support and career guidance | | mobility constraints | |
|---|---|---|---|---|---|---|---|---|
| | (1) | (2) | (3) | (4) | (5) | (6) | (7) | (8) |
| Low closeness | 0.128 | 0.128 | 0.698*** | 0.730*** | 0.003 | -0.032 | 0.116* | 0.184* |
| | (0.085) | (0.127) | (0.079) | (0.119) | (0.074) | (0.111) | (0.064) | (0.096) |
| Medium closeness | -0.005 | 0.049 | 0.703*** | 0.658*** | 0.012 | -0.036 | 0.203*** | 0.274*** |
| | (0.063) | (0.099) | (0.059) | (0.092) | (0.055) | (0.086) | (0.047) | (0.075) |
| High closeness | -0.055 | 0.039 | 0.841*** | 0.841*** | -0.074 | -0.158* | 0.184*** | 0.221*** |
| | (0.062) | (0.095) | (0.057) | (0.089) | (0.054) | (0.083) | (0.046) | (0.071) |
| Female | 0.018 | 0.046 | 0.231*** | 0.226*** | 0.030 | 0.002 | 0.003 | 0.030 |
| | (0.039) | (0.046) | (0.036) | (0.043) | (0.034) | (0.040) | (0.029) | (0.034) |
| Low c. * female | | -0.003 | | -0.054 | | 0.063 | | -0.121 |
| | | (0.165) | | (0.153) | | (0.143) | | (0.124) |
| Medium c. * female | | -0.095 | | 0.075 | | 0.084 | | -0.121 |
| | | (0.129) | | (0.120) | | (0.112) | | (0.097) |
| High c. * female | | -0.163 | | 0.0001 | | 0.146 | | -0.065 |
| | | (0.125) | | (0.116) | | (0.109) | | (0.94) |
| Children | 0.217*** | 0.214*** | -0.022 | -0.022 | -0.109*** | -0.107*** | 0.084** | 0.084** |
| | (0.043) | (0.043) | (0.040) | (0.040) | (0.038) | (0.038) | (0.033) | (0.033) |
| Co - living | -0.323*** | -0.321*** | 0.104* | 0.104* | 0.196*** | 0.195*** | -0.164*** | -0.164*** |
| | (0.057) | (0.057) | (0.053) | (0.053) | (0.050) | (0.050) | (0.043) | (0.043) |
| Postdoc | 0.154*** | 0.157*** | 0.065 | 0.065 | -0.020 | -0.023 | 0.132*** | 0.135*** |
| | (0.049) | (0.049) | (0.045) | (0.045) | (0.042) | (0.042) | (0.037) | (0.037) |
| Prof | 0.034 | 0.040 | 0.127*** | 0.126*** | -0.006 | -0.012 | -0.283*** | -0.278*** |
| | (0.053) | (0.053) | (0.049) | (0.049) | (0.046) | (0.046) | (0.040) | (0.040) |
| Constant | 0.149** | 0.132** | -0.452*** | -0.449*** | -0.083 | -0.066 | 0.111** | 0.095** |
| | (0.061) | (0.063) | (0.057) | (0.059) | (0.053) | (0.055) | (0.046) | (0.047) |
| Obs. | 1,993 | 1,993 | 1,993 | 1,993 | 1,993 | 1,993 | 1,993 | 1,993 |
| $R^2$ | 0.038 | 0.039 | 0.181 | 0.181 | 0.013 | 0.014 | 0.085 | 0.086 |
| Adjusted $R^2$ | 0.034 | 0.034 | 0.178 | 0.177 | 0.009 | 0.009 | 0.081 | 0.081 |

*Note: This table is similar to Table S4; ***$p < 0.1$; **$p < 0.05$; *$p < 0.01$; Standard errors in parentheses.*
