# Supplementary figures and images for: Does love in the ivory tower fix the leaky pipeline? How academia’s homogamous relationships shape careers
Source: PLoS One. 2026 Mar 25;21(3):e0344105. doi: 10.1371/journal.pone.0344105 (PMC13016316; doi:10.1371/journal.pone.0344105)

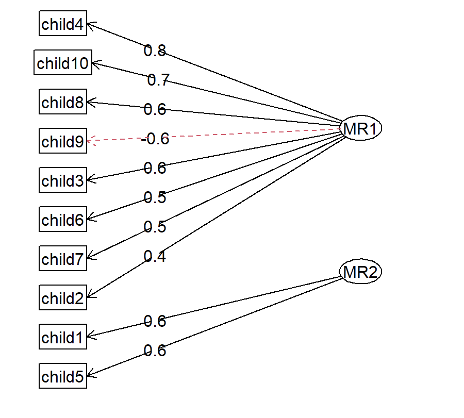

Supplement: S1 Fig — (TIFF) [file pone.0344105.s006.tiff]

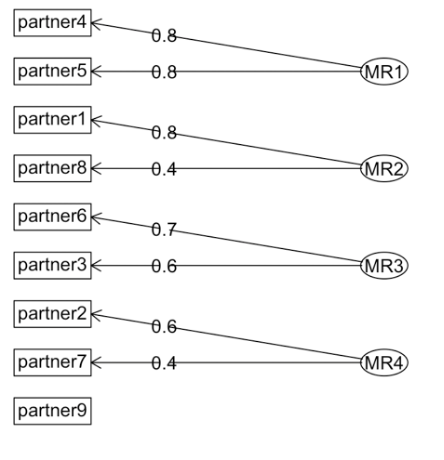

Supplement: S2 Fig — (TIFF) [file pone.0344105.s007.tiff]
